# In Silico Identification of SOX1 Post-Translational Modifications Highlights a Shared Protein Motif

**DOI:** 10.3390/cells9112471

**Published:** 2020-11-13

**Authors:** Azaz Ahmad, Stephanie Strohbuecker, Claudia Scotti, Cristina Tufarelli, Virginie Sottile

**Affiliations:** 1 School of Medicine, The University of Nottingham, Nottingham NG7 2RD, UK; azazkhan47@gmail.com (A.A.); StephanieStrohbuecker@web.de (S.S.); 2 Department of Molecular Medicine, The University of Pavia, 27100 Pavia, Italy; claudia.scotti@unipv.it; 3 Department of Genetics and Genome Biology/Leicester Cancer Research Centre, The University of Leicester, Leicester LE2 7LX, UK; cristina.tufarelli@leicester.ac.uk

**Keywords:** SOX1, post-translational modification, in silico, transcription factors, regulatory motifs

## Abstract

The transcription factor SOX1 is a key regulator of neural stem cell development, acting to keep neural stem cells (NSCs) in an undifferentiated state. Postnatal expression of Sox1 is typically confined to the central nervous system (CNS), however, its expression in non-neural tissues has recently been implicated in tumorigenesis. The mechanism through which SOX1 may exert its function is not fully understood, and studies have mainly focused on changes in SOX1 expression at a transcriptional level, while its post-translational regulation remains undetermined. To investigate this, data were extracted from different publicly available databases and analysed to search for putative SOX1 post-translational modifications (PTMs). Results were compared to PTMs associated with SOX2 in order to identify potentially key PTM motifs common to these SOXB1 proteins, and mapped on SOX1 domain structural models. This approach identified several putative acetylation, phosphorylation, glycosylation and sumoylation sites within known functional domains of SOX1. In particular, a novel SOXB1 motif (xKSExSxxP) was identified within the SOX1 protein, which was also found in other unrelated proteins, most of which were transcription factors. These results also highlighted potential phospho-sumoyl switches within this SOXB1 motif identified in SOX1, which could regulate its transcriptional activity. This analysis indicates different types of PTMs within SOX1, which may influence its regulatory role as a transcription factor, by bringing changes to its DNA binding capacities and its interactions with partner proteins. These results provide new research avenues for future investigations on the mechanisms regulating SOX1 activity, which could inform its roles in the contexts of neural stem cell development and cancer.

## 1. Introduction

SOX1 is a transcription factor mainly known as an early neural stem cell marker, which has also been implicated in tumorigenesis outside of its neural context. Studies have demonstrated that the SOX family of genes can serve as either tumour suppressor genes or oncogenes in different cancers types [1,2]. Autoantibodies to SOX1 are common in small-cell lung carcinoma (SCLC) and serve as a serological tumour marker for SCLC, while the role of a SOX1-related autoimmune response in SCLC is still elusive [3,4]. SOX1 function in neural stem cell maintenance has been studied widely [5,6], but little is known about the role of SOX1 in cancer.

In addition to mechanisms regulating gene expression at a transcriptional and epigenetic level, molecular function can also be regulated by modulating the properties of the encoded protein through different types of post-translational modifications (PTMs) [7]. There are several PTMs that can occur in a protein sequence depending on the identity of its amino acids. There is currently no information with regards to PTMs affecting SOX1 protein function in either neural stem cells or cancer. In this study, an in-silico analysis was performed [8] on the SOX1 protein sequence to identify PTMs using different bioinformatics tools and data sources, in order to provide new information hinting to potential mechanisms regulating SOX1 function in different cellular contexts.

## 2. Materials and Methods

PTM identification: Evidence of potential PTM residues in the human SOX1 protein was surveyed using the protein name human SOX1 (ACC#O00570) or SOX1 amino acid sequence in a FASTA format. The PhosphoSitePlus® database [9] was searched to identify existing experimentally verified data about SOX1 PTMs (http://www.phosphosite.org/homeAction.do). The NetPhos 3.1 server (http://www.cbs.dtu.dk/services/NetPhos) was used to predict phosphorylation sites at serine, threonine and tyrosine residues for SOX1 [10]. YinOYang 1.2 (http://www.cbs.dtu.dk/services/YinOYang) was used for prediction of YinOYang sites [11,12]. GSP-SUMOv2 (http://sumosp.biocuckoo.org) [13] and JASSAv4 (http://www.jassa.fr) [14] were used as sumoylation prediction tools.

Multiple sequence alignment (MSA) tools: Protein BLAST [15] was used to identify homologous sequences for the Human SOX1 protein in the refseq collection database (refseq_protein) [16], selecting Mouse (NP_033259), Chimpanzee (XP_016781055), Rabbit (XP_002724136), Whale (XP_004273645), Monkey (XP_017357713), Dog (XP_849239), and Chicken (NP_989664) sequences to perform multiple sequence alignment (MSA) [17] using Clustal-Omega from the EMBL-EBI resources [18].

Consensus motif search: ScanProsite [19] was used to scan for the SOXB1 consensus motif XKSExSxxP for matches against other proteins, selecting UniProtKB and searching against human proteins only, excluding the option of splice variants. The result obtained was matched against UniProt protein entries and was analysed by retrieving the Gene ontology (molecular) in the UniProt database [20]. InterPro (https://www.ebi.ac.uk/interpro/search/sequence/) and Eukaryotic Linear Motif (ELM, http://elm.eu.org/) databases were used to search against recognised protein motifs.

SNPs analysis within SOX1 coding locus: NCBI-dbSNP [21] and Uniprot feature searches [20] were used for SNP analysis.

IBS Illustrator for Biological Sequences: The IBS webserver was used for schematic illustration of SOX1 protein sequence figures [22].

Protein disorder and modelling: SOX1 disorder was evaluated by IUPred and ANCHOR2 [23]. Protein domain modelling was performed by Modeller [24] for SOX1 HMG, residues 49-120, using the structure of SOX2 HMG transcription factor as a template (PDB ID: 1o4x). I-TASSER [25] was instead used for modelling of SOXp (residues 129-194) and the C-terminal (residues 263-366) domains, based on its excellent performance in all recent CASP competitions [26,27]. The structure for intrinsically disordered regions (IDRs) was build using the same strategy as for structured protein [28], and the secondary structure prediction was based on the PSSpred algorithm, a combination of 7 neural network predictors from different profile data and parameters [29,30]. All models were geometry optimized by Phenix [31] and validated by ProSA Web [32], Verify 3D [33] and Rampage [34]. PTMs were modelled by Vienna-PTM 2.0 [35] and molecules visualised by Pymol [36].

## 3. Results

### 3.1. Survey of PTMs of the SOX1 Protein

**Figure 1:**
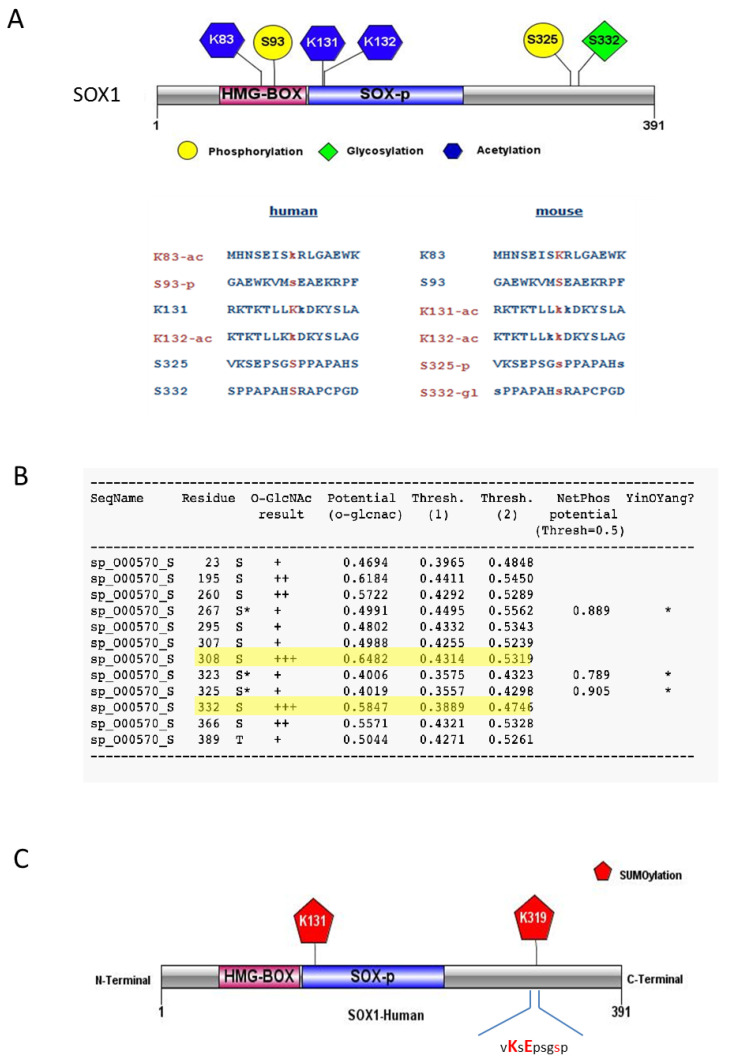
Post-translational modifications (PTMs) predicted for the SOX1 protein. (**A**) Schematic representation of different PTM types identified from the PhosphoSitePlus database with multiple sequence alignment for both human and mouse SOX1 protein showing validated (red) and predicted (blue) PTM residues. Phosphorylation (-p), Acetylation (-ac) and Glycosylation (-gl). (**B**) Glycosylation (O-GlcNAc), phosphorylation (-p) and predicted residues with YinOYang effect (asterisk) from the YinOYang 1.2 Server. (**C**) Sumoylated residues within SOX1 predicted by GSP-SUMOv2.0 and JASSAv4 analysis tools. Diagrams in (**A**) and (**C**) generated using the IBS illustrator webserver [22].

Databases from PhosphoSitePlus, YinOYang 1.2 server and NetPhosK 3.1 server were surveyed for predicted PTMs. This analysis showed the presence of a variety of putative sites on SOX1, such as phosphorylation, sumoylation, acetylation and glycosylation (Figure 1) (Figures S1–S3). Predicted PTM sites were primarily found within functional SOX1 domains, such as the HMG-domain and C-terminal region [9] (Figure 1A). Both predicted and experimentally verified acetylation of lysine (K) residues was found at HMG-BOX and SOXp domain of SOX1 (Figure 1A). Residues K83 and K132 in human SOX1 were experimentally verified to be acetylated, while K131 was predicted to be acetylated (Figure 1). K131 and K132 acetylation was also experimentally verified in mouse SOX1 (Figure 1A) [9].

PhosphoSitePlus indicated phosphorylation sites at serine (S) residues at positions S93 and S325 (Figure 1A), that were also predicted by NetPhosK server 3.1, with phosphorylation potential scores of 0.829 and 0.904, respectively (Figure 1B; Figures S1–S3). Site S93 was the only experimentally verified phosphorylation site reported for the human SOX1 protein, found phosphorylated in tumour tissue affected by ischemia [37]. S93 is found within the HMG-Box of SOX1 (Figure 1A), a DNA binding domain whose phosphorylation might be crucial to SOX1 DNA binding and transcription factor activities. Residue S325 is present within the C-terminal region of SOX1, which is known for its transcriptional activation [38]. PhosphoSitePlus reported S325 as an experimentally verified phosphorylated site in mouse SOX1 [39,40], suggesting it might be phosphorylated in human SOX1 as well.

The survey of glycosylation sites within the SOX1 protein was performed using YinOYang 1.2 server tools [11,12], and indicated SOX1 residues S325, S332 and S308 (Figure 1A,B) were predicted to be glycosylated. S332 was also experimentally verified in mouse SOX1 by PhosphositePlus [39] (Figure 1A). A predicted Yin-Yang effect [9,39] was found at residue S325, which was also predicted to be phosphorylated (Figure 1B), while residues S308 and S332 showed high probability of O-GlcNAc but much less probable Yin-Yang effect (Figure 1B, highlighted in yellow).

Sumoylation analyses performed with GSP-SUMOv2.0 and JASSAv4 showed two potential sites for human SOX1 sumoylation, at residues K131 and K319 (Figure 1C). K319 is present within the C-terminal domain, and this domain is known for the transactivation ability of SOX1. Residue K131 was predicted sumoylated and also acetylated, and was found located close to the DNA binding domain of SOX1 (HMG-domain), where PTMs may exert a regulatory role for SOX1 DNA binding activity.

### 3.2. Phosphorylation-Dependent Sumoylation Sites Within SOX1

The canonical sumoylation consensus motif in a protein sequence is ψKxE, where ‘ψ‘ can be any hydrophobic amino acid such as A, I, L, M, P, F, V or W, while ‘x’ can be any amino acid [41]. In SOX1, residue K319 was found to lie within the sumoylation consensus motif _318_vKsE_321_ (Figure 1C). The K319 sumoylation consensus motif is in close proximity to S325 (_318_vKsEpsgSp_326_), which is a predicted phosphorylation site (Figure 1A,C). Since studies have shown that phosphorylation may regulate sumoylation of a substrate [41], further analysis was carried out on this specific motif (_318_VKSEPSGSP_326_) containing sumoylation and phosphorylation sites, to investigate whether it could provide additional information about the regulation of SOX1 function.

### 3.3. Identification of a New SOXB1 Consensus Motif Within the SOX1 Protein

**Figure 2:**
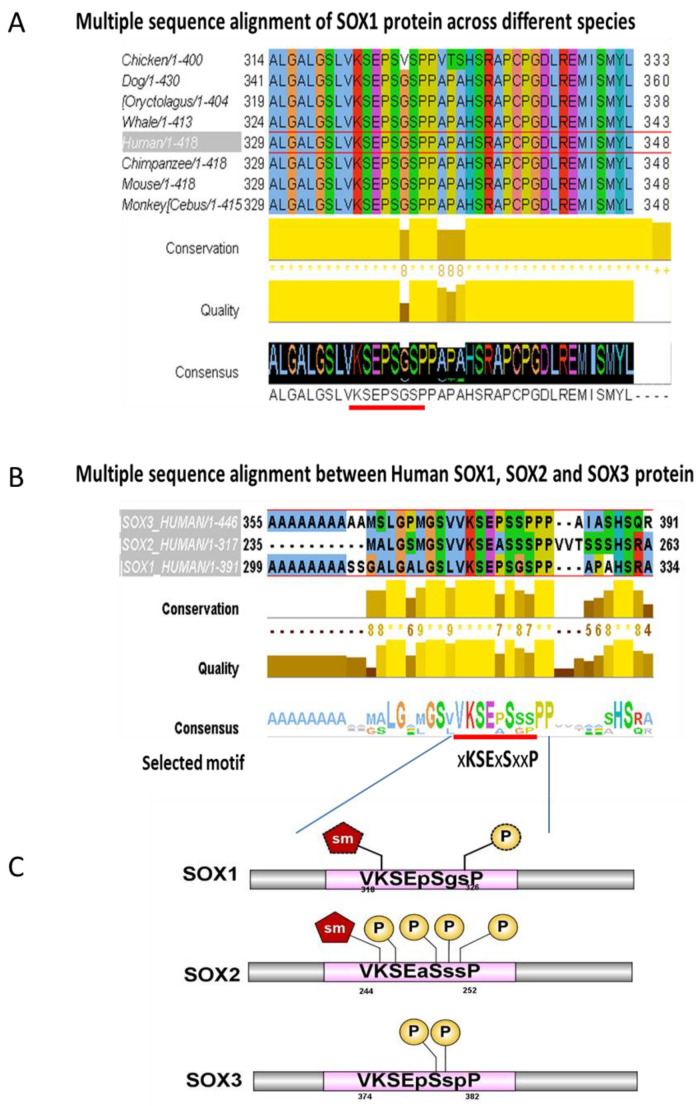
Multiple Sequence alignment of SOX1 protein. (**A**) Multiple Sequence alignment (MSA) for SOX1 protein focusing on the highly conserved motif _318_VKSEPSGSP_326_ (underlined in red). (**B**) MSA between members of the SOXB1 subgroup, focusing on the consensus motif named as ‘SOXB1 consensus motif’ used to further search protein databases. Figure generated using the EMBL-EBI resources, ClustalOmega alignment output page [17]. (**C**) Predicted PTMs within SOXB1 consensus motif. Conserved amino acids are shown in capital letters. P; Phosphorylation, sm; Sumoylation. IBS illustrator was used to produce the diagram [22].

Evolutionary conserved regions generally point to key functional parts of a protein. Multiple Sequence Alignment (MSA) of SOX1 across different species revealed that the region _318_VKSEPSGSP_326_ is highly conserved across different species (Figure 2A). The transcription factors SOX1, SOX2 and SOX3 belong to the SOXB1 sub family, sharing high sequence similarities and relatively similar expression patterns [6]. MSA investigation of the homology in the _318_VKSEPSGSP_326_ region among transcription factors belonging to the SOXB1 sub-family showed that it is highly conserved across this sub-family (Figure 2B). Based on the level of conservation, the consensus sequence xKSExSxxP was identified as SOXB1 consensus motif (Figure 2C), where ‘x’ can be any amino acid.

The consensus motif was not recognised to be associated with any specific protein family listed in the InterPro platform, while a search in the Eukaryotic Linear Motif (ELM) database did not yield a full homology but only returned partial matches to (i) a CK1 phosphorylation site (‘SEPSGSP’, with a probability score of 1.70e–02), (ii) to a GAG attachment site (‘(E)PSGS’, with a probability of 1.92e–02) and (iii) to a SUMO-1 modification site (‘VKSE’, with a probability score of 1.91e–03) (Table S1).

**Figure 3:**
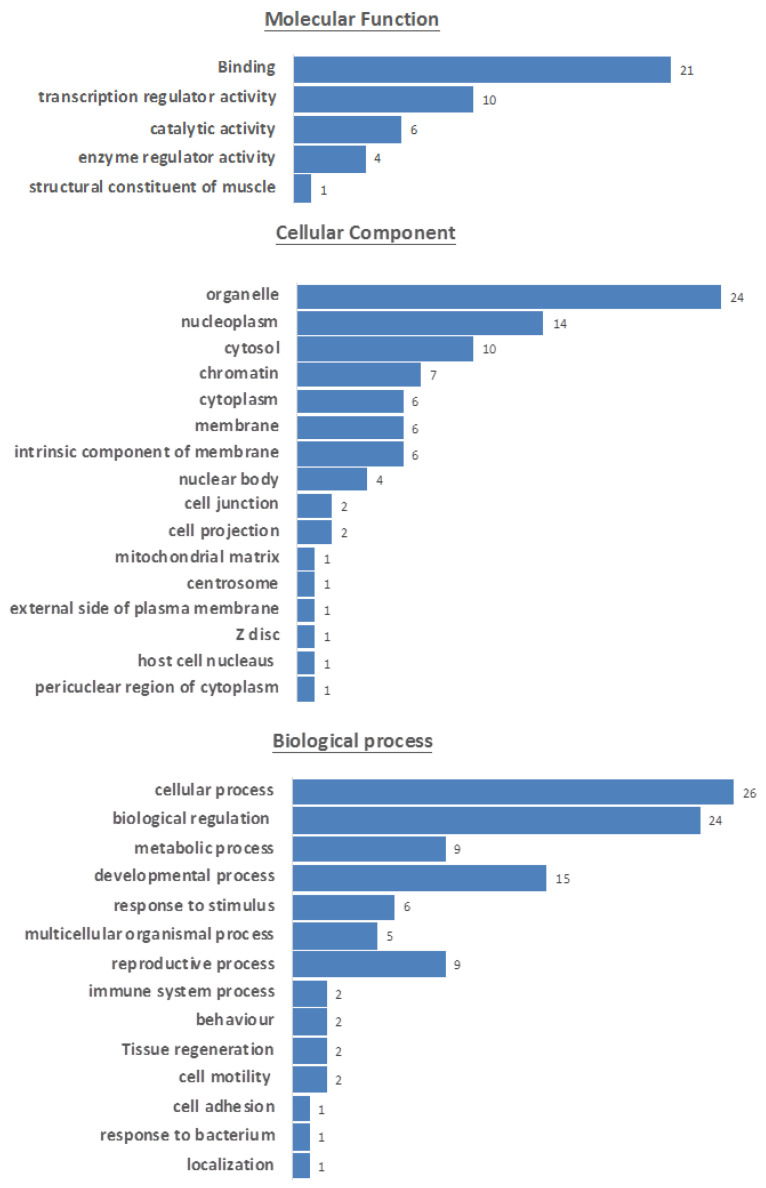
Gene ontology information of proteins sharing the SOXB1 motif highlighting involvement in common processes represented by blue horizontal bars, based on information (Table S3) retrieved from UniProt [20].

To further investigate whether the SOXB1 consensus motif is present in other unrelated proteins, a collection of human protein motifs from the ScanProsite database was surveyed. Interestingly, the SOXB1 consensus motif highlighted in Figure 2 was found to be shared by 32 unrelated proteins (Table S2). Gene ontology analysis of these proteins (Figure 3) showed that the majority are transcription regulators acting as transcriptional activators/repressors with sequence-specific DNA binding, and are involved in developmental processes [42].

The level of conservation assessed in a subset of proteins sharing this motif (Figure S4) showed conservation, supporting the hypothesis of a functional role.

### 3.4. SOX1 Variants Triggering Changes to PTMs

**Figure 4:**
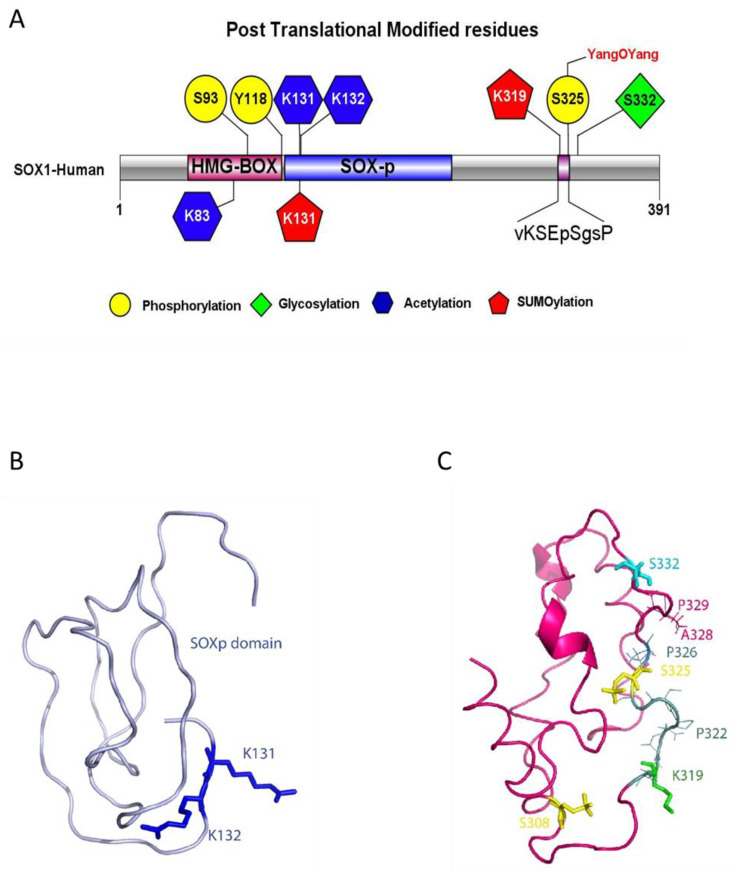
Models of SOX1 PTMs and structure. (**A**) SOX1 structure with predicted post translational modification sites and SOXB1 consensus motif sequence. The IBS illustrator was used to generate the diagram [22]. (**B**) SOXp domain model (residues 129–194). In blue: acetylated lysine residues K131 and K132. Residue K131 is also predicted to be sumoylated. (**C**) C-terminal domain model (residues 263–366). Sticks represent PTMs. In yellow: residues S308 and S325 are predicted to be phosphorylated. In green: residue K319 is predicted to be sumoylated. In light blue: residue S332 is predicted to be glycosylated. Thin lines show the residues forming the SOXB1 motif beyond S325 and V318. Labels are also provided for SNPs locations (P322, P326, A328, P329). Models in (**B**) and (**C**) were generated using I-TASSER [25], geometry optimized by Phenix [31] and validated by ProSA Web [32], Verify 3D [33] and Rampage [34]. PTMs were modelled by Vienna-PTM 2.0 [35] and molecules visualised by Pymol [36].

An analysis was conducted to identify Single Nucleotide Polymorphisms (SNPs) within the SOX1 gene locus causing mutations in the SOX1 coding region. NCBI-dbSNP and Uniprot feature searches [21] highlighted many SNPs present at the SOX1 coding locus. SNPs triggering amino acid changes in the regions of interest containing HMG-domain, SOXp and C-terminal region of SOX1 protein were selected (Figure 4A). This analysis revealed that SNP rs1419694769 (A > T), which causes a missense mutation at K132 (K > E), leads to loss of acetylation at this crucial position (Table S4). Other relevant variants identified were rs1389486372, rs1178459411, rs1294049725, rs1161821962, rs1382937308 and rs1362549824. These SNPs are all linked to amino acid sequence changes within the SOXB1 consensus motif and in its close proximity, triggering loss/changes to the PTMs identified at this crucial motif. Moreover, they imply in three cases out of five the replacement of a Pro residue, which has relevant structural roles in loop rigidification and binding sites bracketing [43].

### 3.5. SOX1 Structural Modelling

Analysis of SOX1 sequence by IUPred and ANCHOR2 highlighted several regions with a high intrinsic disorder (Figure S5A). An attempt was made to model the full-length protein structure with I-TASSER, and the final structure evidenced three folded domains separated by disordered sequences: the HMG Box (residues 49–120), the SOXp (residues 129–194) and the C-terminal domains (residues 263–366), which turned out to cover all of the identified PTMs (Figure S5B). Further modelling work was then performed on each of these three individual domains. In particular, the model for the C-terminal domain is to be considered as a structurally plausible solution among the ensemble of structures potentially assumed by the IDR. In this respect, the validation of the model provided by three different servers (Table S5) confirmed its reliability. The α-helix predicted to include residues 288–290 has a low confidence score (<1), while the one including the REMIS sequence (residues 342–346) has confidence scores of eight or nine for each residue (Figure 4B).

**Figure 5:**
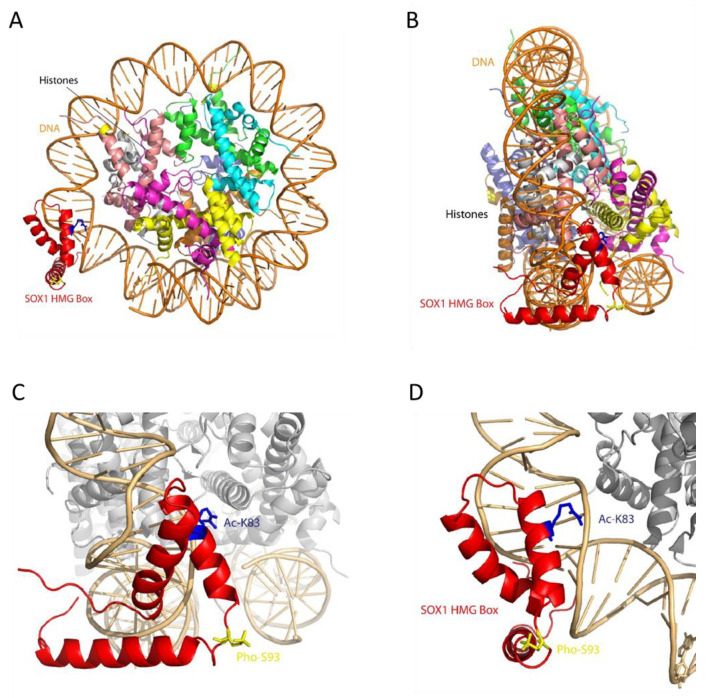
Structural model of SOX1 HMG Box domain (residues 49–120) generated by Modeller [24], using the structure of SOX2 HMG transcription factor as a template (PDB ID: 1o4x). (**A**) Model of SOX1 interaction with a nucleosome, based on SOX2 (PDB ID: 67TB). (**B**) 90° rotation of (**A**). (**C**) Magnification of the SOX1 DNA binding domain (HMG box). (**D**) Rotation by 90 degrees of (**C**). In red: SOX1 HMG Box domain. In blue: acetylated K83. In yellow: phosphorylated S93.

The models of the three domains were then used to introduce phosphorylation and acetylation in the predicted positions by VIENNA-PTM 2.0, while, for other PTMs, residues involved were highlighted on the models and visualised by Pymol. The three-dimensional models of the SOXp domain and of the C-terminal domain (Figure 4 panels B and C, respectively) with the locations of the predicted PTMs and of the novel SOXB1 motif (identified by residues shown as sticks) show the PTMs all clustered together, especially in the C-terminal domain, where also several SNPs map, suggesting a close functional interaction of the relevant residues and modifications. SOX1 HMG Box is a well-characterized DNA binding domain, which, from the structural point of view, includes three α-helices, numbered from one to three, that are highly conserved throughout the SOX family of transcription factors [44]. PDB ID 6T7B describes the structure of human SOX2 transcription factor (HMG box domain) in complex with a nucleosome. Superposition of the highly homologous SOX1 HMG Box domain model (RMSD: 0.20 Å) showed that phosphorylation of residue S93 affects a position located in the loop comprised between the second and third α-helices, while acetylation affects K83, a residue located very close to the DNA molecule and potentially interacting with it (Figure 5). Interestingly, the only three amino acid differences which distinguish the SOX1 and SOX2 HMG Box sequences (positions 91, 92 and 95) [45] are the main components of the five-residue connection loop between α-helices two and three. This sequence tends to be specific to each SOX family member. Its location, pointing outside the nucleosome, suggests it may be involved in the recruitment of specific molecular partners.

## 4. Discussion

The PTM data uncovered in this study (summarised in Figure 4) strongly suggest that SOX1 might be regulated at the post-translational level, which could impact its function. Depending on its post-translational modifications, a transcription factor might change its DNA binding activities and interactions with partner proteins within transcription regulatory complexes, consequently altering its function [45,46]. This paradigm is well exemplified by SOX2, a SOX1-related transcription factor belonging to the same SOXB1 subfamily, and given SOX1 and SOX2 show functional redundancy [47], mechanisms regulating SOX2 provide useful information on the potential role of PTMs in SOX1 function. Liu et al. have shown that different PTMs confer SOX2 variable functions that regulate stem cell pluripotency and differentiation [48]. SOX2 sumoylation at K247 impairs its binding to the Fgf4 enhancer, which results in negative regulation of SOX2 transcriptional activities [49]. In mouse embryonic stem cells, interaction of SOX2 with the nuclear export machinery by acetylation at K75 within the HMG domain retains SOX2 inside the nucleus to regulate its target genes [50]. Consistent with this, the present study indicates that SOX1 is predicted to harbour sites of sumoylation at K319 and acetylation at K83.

The HMG domain of SOX1 is an evolutionarily conserved and functionally important region that binds to specific DNA sequences to bring conformational changes within chromatin structure [51]. The activity of the HMG Box domain is both to bind to and to bend DNA, and this happens thanks to residue M59, absolutely conserved throughout the SOX family of transcription factors, which indents within the DNA double helix [44]. The predicted post-translationally modified residues identified at K83 (acetylated) and S93 (phosphorylated) lie within the HMG domain of SOX1, making these modifications significant to SOX1 function. In fact, evaluation of the HMG box SOX-1 domain model in the structural context of the nucleosome suggests that these PTMs could interfere with its direct interaction with nucleosomal DNA (Ac-K83) or with other molecular partners (Pho-S93).

Another important SOX1 domain is SOXp, as binding site for the nestin neural enhancer [52] which is in close proximity to the HMG domain. It has been documented that binding of SOX2 to the nestin enhancer upregulates the expression of Nestin [52]. In SOX1, the acetylated and structurally adjacent residues K131 and K132 might similarly influence SOX1 binding to the nestin enhancer. K131 is also predicted as a sumoylation site, therefore interplay between acetylation and sumoylation is likely to occur at this site. The interplay between sumoylation and acetylation at K131 might be involved in regulating the transcriptional and DNA binding activity of SOX1, in line with evidence of cross-talk between sumoylation and acetylation regulating transcription and DNA binding activity of the tumour suppressor p53 [53].

Similarities between the PTMs predicted in SOX1 and those reported for SOX2 highlight the need to further investigate whether they may affect protein function in a comparable way. Interestingly, the SOXB1 motif identified in this study is not unique to the SOXB1 family of proteins, but is in fact present among other unrelated proteins, the majority of which are transcription factors involved in biological processes such as transcription regulation, DNA binding and transcription factor activity. The sequence structure of the SOXB1 motif identified in SOX1 fits with reports in the literature of sumoylation motifs being adjacent to sites of proline-directed phosphorylation [41]. Indeed, the SOXB1 motif identified in SOX1 includes the predicted phosphorylation site S325, which precedes proline residue P326, and includes the predicted sumoylated site K319. This suggests that the SOXB1 motif within SOX1 is a phosphorylation-dependent sumoylation motif. The majority of transcriptional regulators, such as heat-shock factors (HSFs) and transcription factors such as GATA-1 and myocyte enhancer factor 2 contain a phosphorylation-dependent sumoylation motif [41]. Phosphorylation-dependent sumoylation has been shown to repress transactivation capacities of HSF family proteins [41]. The SOXB1 motif identified here is conserved among SOX1 and SOX2. Since phospho-sumoyl switches have been reported at a similar conserved region of SOX2 [54] and sumoylation within this region was seen to affect SOX2 transcriptional activity [55], our findings suggest that SOX1 the transcription factor function may be regulated by phosphorylation-dependent sumoylation at this specific motif.

From the structural point of view, SOX-1 includes several predicted disordered amino acid regions, especially at the C-terminal in the HMG domain. It is reasonable to assume that the full-length protein reaches the correct functional conformation upon specific partner binding and that PTMs can act either as activity or affinity modifiers. Selection of specific protein partners is therefore likely to play a major role in regulating and generating cell and developmental stage-specific functions for SOX transcription factors [56]. There is no information currently available regarding the details of the tertiary structure of SOX1, but the fact that PTM modification sites are clustered together, especially in the modelled C-terminal domain, suggests that alternative or simultaneous modifications can contribute to switching on and off functions even by a structural hindrance-mediated mechanism and/or by modifying inter-domain interactions.

Changes in the genomic coding sequence that can alter the amino acid sequence may change protein function either through direct structural changes or through alterations in PTMs. Here, evidence for single nucleotide polymorphisms (SNPs) within SOX1 protein was reviewed, in particular for the existence of mutations within the SOXB1 consensus motif, which might affect PTMs in this target region. An SNP causing a missense mutation (K132 > E132) was linked to loss of acetylation at K132, which could affect the DNA binding activity of SOX1 as a transcription factor [52] or its nuclear import [50]. Future mutational analyses experiments will be needed to better evaluate the role of these residues and their link to SOX1 regulation, both as a neural marker and for its role in cancer development. In addition, wider comparative genomic analyses between pathological and control samples could highlight point mutations in upstream sequences, including the promoter region, which could inform on critical regulatory regions.

SOX1 PTMs will need to be experimentally validated in order to clarify the mechanisms regulating SOX1 transcriptional function. For example, SOX1 plays a regulatory role during neuronal cell fate determination and differentiation as it binds to the Hes1 promoter, attenuating Notch signalling that suppresses neurogenesis [57]. The SOX1 HMG and C-terminal domains are both required for interaction with the Hes1 promoter [57]. Therefore, PTMs predicted within these domains could have a possible effect on SOX1 binding and activity at the Hes1 promoter. SOX1 is also known to bind to β-catenin to attenuate Wnt signalling, while dysregulated Wnt signalling has been reported in many cancer types [40,58]. Acetylation of SOX1 at K83, as raised by the present analysis, might be involved in retaining SOX1 inside the nucleus [59]. Further research will determine whether blocking K83 acetylation can modify SOX1 function in neurodevelopment and cancer.

## 5. Conclusions

In conclusion, our analysis has identified a set of putative PTMs within SOX1 suggested to regulate SOX1 function as a transcription factor. Previous studies have shown how the identification of functional regulatory regions through computational analysis has been validated experimentally. For example, putative HMG and POU motifs identified in silico were subsequently confirmed experimentally to exert a functional role in the regulation of DPPA4, a gene that plays an important role in ES cell self-renewal [60]. In-silico analysis can thus help address biological questions more efficiently by identifying new potential regulatory regions. Results presented here will inform future experimental investigations on the differential regulation of SOX1 transcriptional function in neural stem cell fate and in cancer.

## Supplementary Materials

The following are available online at https://www.mdpi.com/2073-4409/9/11/2471/s1, Figure S1: SOX1 PTM result from the analysis with the NetPhosK 3.1 server. Figure S2: Graphical output from the SOX1 PTM analysis with NetPhosK 3.1. Figure S3: Graphical output from the SOX1 PTM analysis with PhosphoPlus. Figure S4: Multiple sequence alignment (MSA) of SOXB1 consensus motif. MSA of SOXB1 consensus motif among different organisms, performed for 5 randomly selected proteins from the list in Table S2 (#5, 7, 8, 13, 23, respectively) analysed with Clustal Omega alignment (EMBL-EBI resources [17]) showing SOXB1 consensus motif region (yellow), positions which have a single, fully conserved residue (‘*’), conservation between groups of strongly similar properties (‘:’), and conservation between groups of weakly similar properties (‘.’). Figure S5: Analysis of SOX1 sequence. (A) Analysis with IUPred and ANCHOR2 highlighted several regions with a high intrinsic disorder. (B) I-TASSER model of full-length SOX1 produced with I-TASSER. Table S1: Results of an Eukaryotic Linear Motif (ELM) database search for the consensus motif ‘VKSEPSGSP‘. Table S2: List of transcription factors sharing the SOXB1 consensus motif (xKSExSxxP) identified using the ScanProsite database. Table S3: Gene ontology information from UniProt for the proteins identified. Table S4: SOX1 SNPs collected from NCBI-dbSNP and Uniprot. Table S5: Statistical report for the 3 validation servers for the 3 modelled domains.
