# Supplementary material for: In Silico Identification of SOX1 Post-Translational Modifications Highlights a Shared Protein Motif
Source: Cells. 2020 Nov 13;9(11):2471. doi: 10.3390/cells9112471 (PMC7696889; doi:10.3390/cells9112471)
Supplement: Supplementary file 1 [file cells-09-02471-s001.pdf]

Figure S1

NetPhos 3.1 Server - prediction results

Technical University of Denmark

```
>sp_O00570_SOX1_HUMAN   391 amino acids
#
# netphos-3.1b prediction results
#
# Sequence                   # x   Context     Score   Kinase    Answer
# -------------------------------------------------------------------
# sp_O00570_SOX1_HUMAN     12 S   TDLHSPGGA   0.963   unsp       YES
# sp_O00570_SOX1_HUMAN     20 T   AQAPTNLSG   0.529   cdc2       YES
# sp_O00570_SOX1_HUMAN     93 S   WKVMSEAEK   0.829   unsp       YES
# sp_O00570_SOX1_HUMAN    118 Y   EHPDYKYRP   0.942   unsp       YES
# sp_O00570_SOX1_HUMAN    126 T   PRRKTKTLL   0.961   unsp       YES
# sp_O00570_SOX1_HUMAN    128 T   RKTKTLLKK   0.877   PKC        YES
# sp_O00570_SOX1_HUMAN    135 Y   KKDKYSLAG   0.971   unsp       YES
# sp_O00570_SOX1_HUMAN    136 S   KDKYSLAGG   0.798   PKA        YES
# sp_O00570_SOX1_HUMAN    172 S   QRLESPGGA   0.996   unsp       YES
# sp_O00570_SOX1_HUMAN    181 Y   AGGGYAHVN   0.791   unsp       YES
# sp_O00570_SOX1_HUMAN    249 Y   PMHRYDMGA   0.530   unsp       YES
# sp_O00570_SOX1_HUMAN    257 S   ALQYSPISN   0.722   unsp       YES
# sp_O00570_SOX1_HUMAN    260 S   YSPISNSQG   0.560   cdc2       YES
# sp_O00570_SOX1_HUMAN    262 S   PISNSQGYM   0.633   DNAPK      YES
# sp_O00570_SOX1_HUMAN    265 Y   NSQGYMSAS   0.731   unsp       YES
# sp_O00570_SOX1_HUMAN    267 S   QGYMSASPS   0.889   unsp       YES
# sp_O00570_SOX1_HUMAN    269 S   YMSASPSGY   0.848   unsp       YES
# sp_O00570_SOX1_HUMAN    271 S   SASPSGYGG   0.950   unsp       YES
# sp_O00570_SOX1_HUMAN    273 Y   SPSGYGGLP   0.606   unsp       YES
# sp_O00570_SOX1_HUMAN    316 S   GALGSLVKS   0.742   PKC        YES
# sp_O00570_SOX1_HUMAN    320 S   SLVKSEPSG   0.749   unsp       YES
# sp_O00570_SOX1_HUMAN    323 S   KSEPSGSPP   0.788   unsp       YES
# sp_O00570_SOX1_HUMAN    325 S   EPSGSPPAP   0.904   unsp       YES
# sp_O00570_SOX1_HUMAN    332 S   APAHSRAPC   0.594   PKG        YES
# sp_O00570_SOX1_HUMAN    370 S   SRLHSLPQH   0.822   unsp       YES
# sp_O00570_SOX1_HUMAN    385 T   GVNGTVPLT   0.705   PKC        YES
#
    MYSMMMETDLHSPGGAQAPTNLSGPAGAGGGGGGGGGGGGGGGAKANQDR   #    50
    VKRPMNAFMVWSRGQRRKMAQENPKMHNSEISKRLGAEWKVMSEAEKRPF   #   100
    IDEAKRLRALHMKEHPDYKYRPRRKTKTLLKKDKYSLAGGLLAAGAGGGG   #   150
    AAVAMGVGVGVGAAAVGQRLESPGGAAGGGYAHVNGWANGAYPGSVAAAA   #   200
    AAAAMMQEAQLAYGQHPGAGGAHPHAHPAHPHPHHPHAHPHNPQPMHRYD   #   250
    MGALQYSPISNSQGYMSASPSGYGGLPYGAAAAAAAAAGGAHQNSAVAAA   #   300
    AAAAAASSGALGALGSLVKSEPSGSPPAPAHSRAPCPGDLREMISMYLPA   #   350
    GEGGDPAAAAAAAAQSRLHSLPQHYQGAGAGVNGTVPLTHI            #   400
%1  ...........S.......T..............................   #    50
%1  ..........................................S.......   #   100
%1  .................Y.......T.T......YS..............   #   150
%1  .....................S........Y...................   #   200
%1  ................................................Y.   #   250
%1  ......S..S.S..Y.S.S.S.Y...........................   #   300
%1  ...............S...S..S.S......S..................   #   350
%1  ...................S..............T......
```

Figure S2

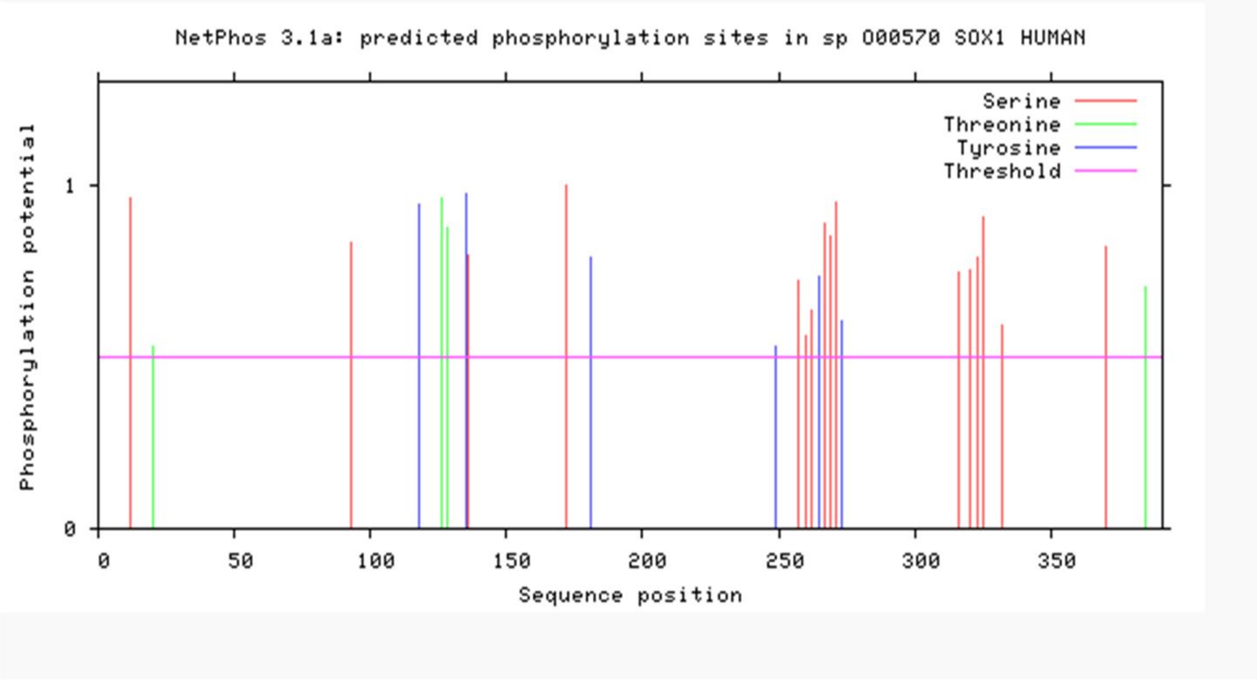
NetPhos 3.1a: predicted phosphorylation sites in sp O00570 SOX1 HUMAN
Serine
Threonine
Tyrosine
Threshold
Phosphorylation potential
1
0
0
50
100
150
200
250
300
350
Sequence position

Figure S3

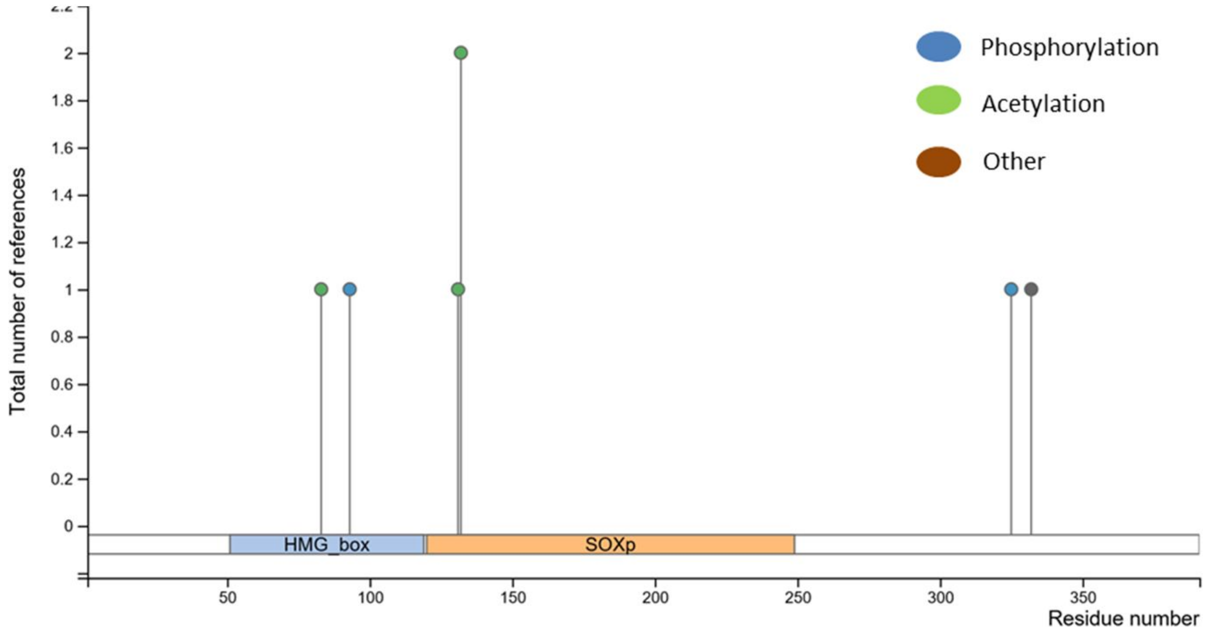

Total number of references
2
1.8
1.6
1.4
1.2
1
0.8
0.6
0.4
0.2
0
Phosphorylation
Acetylation
Other
HMG_box
SOXp
50
100
150
200
250
300
350
Residue number

Figure S4

Uniprot ID		Organism	Amino acid start position		Amino acid end position
**Chromodomain-helicase-DNA-binding protein 7 (CHD7)**					
Q9P2D1	CHD7_HUMAN	Human	2211	ASSVKNELKGVEVGADTGSKSISEKGSEEDEEEKLEDDDKSEESSQPEAGAVSRGKNFDE	2270
A2AJK6	CHD7_MOUSE	Mouse	2200	ASSVKGELKGVEGSADPGSKSVSEKGSEEDEEEKLEDDDKSEESSQPEAGAVSRGKTFDE	2259
Q06A37	CHD7_CHICK	Opossum	2213	TGSVKCELKDIEMSTDVDPKSISEKGSEEDEEEKLDDDDKSEESSQPEAGAVSQGKNFDE	2272
G3R983	G3R983_GORGO	Gorilla	2211	ASSVKNELKGVEVGADTGSKSISEKGSEEDEEEKLEDDDKSEESSQPEAGAVSRGKNFDE	2270
				:.*** ***.:* .:* . **:*************:*****************:**.***	
**Erythroid membrane-associated protein (ERMAP)**					
Q96PL5	ERMAP_HUMAN	Human	387	FSGPLRPFFEPCLHDGGKNTAPLVICSELHKSEESIVPRPEGKGHANGDVSLKVNSSLLP	446
Q9JLN5	ERMAP_MOUSE	Mouse	478	FSSPLRPFFEPCLHDEGKNTAPLIICTELQKSEESIVPKQEGKDRANGDVSLKMNPSLLS	537
F6X7V8	F6X7V8_CANLF	Dog	477	FSGPLRPFFEPCLHDGGKNTAPLIICSELQKSEPPTGPKPEEKVHANGDVAMTVDPSLLP	536
G3QWV0	G3QWV0_GORGO	Gorilla	387	FSGPLRPFFEPCLHDGGKNTAPLVICSELHKSEESTVPKPEGKGHANGDVSLKVNSSLLP	446
				**.************ *******:**:**:*** *: * * :*****::.:: ***	
**Forkhead box protein D3 (FOXD3)**					
Q9UJU5	FOXD3_HUMAN	Human	361	AGTTASLIKSEPSARPSFSIENIIGGGPAAPGGSAVGAGVAG---GTGGSGGGSTAQSFL	417
Q61060	FOXD3_MOUSE	Mouse	346	AGT-TSLIKSEPSARPSFSIENIIGAGSAAPGGSAGGGGSGGGAGGGGGSGGGGGAQSFL	404
P79772	FOXD3_CHICK	Chicken	306	----GSIVKSEPSSRPSFSIENIIGGPAAS--------------------SAPSAQTFL	340
H2R911	H2R911_PANTR	Chimpanzee	361	AGTTASLIKSEPSARPSFSIENIIGGGPAAPGGSAAGAGVAG---GTGGSGGGSTAQSFL	417
				*::*****:***********. *: .. **:**	
**N-myc proto-oncogene protein (MYCN)**					
P04198	MYCN_HUMAN	Human	339	VESEDAPPQKKIKSEASPRPLKSVIPPKAKSLSPRNSDSEDSERRRNHNILERQRRNDLR	398
P03966	MYCN_MOUSE	Mouse	337	VESEDAPPQKKIKSEASPRPLKSVVPAKAKSLSPRNSDSEDSERRRNHNILERQRRNDLR	396
P18444	MYCN_CHICK	Chicken	317	MESEDVPPQKKLKAE-VPRPVKPMIQPKSKSSSPRNSDSEDSERRRNHNILERQRRNDLR	375
G3QQZ3	G3QQZ3_GORGO	Gorilla	339	MESEDAPPQKKIKSEASPRPLKSVIPPKAKSLSPRNSDSEDSERRRNHNILERQRRNDLR	398
				:****.*****:*:* ***:* :: *:** *****************************	
**Signal-induced proliferation-associated 1-like protein 2 (SIPA1L2)**					
Q9P2F8	SI1L2_HUMAN	Human	1380	PGSMSKPYHRQGAVNKYVIGWKKSEGSPPPEEPEVTECPGMYSEMDVMSTATQHQTVVGD	1439
Q80TE4	SI1L2_MOUSE	Mouse	1380	PGAVTKPYHRQGAANKYVIGWKKSEGSPPPEEPEVTECPRIYGEMDIMSTATQHPAVVGD	1439
G3QET1	G3QET1_GORGO	Gorilla	1380	PGSMSKPYHRQGAVNKYVIGWKKSEGSPPPEEPEVTECPGMYSEMDVMSTATQHQTVVGD	1439
G1SZ09	G1SZ09_RABIT	Rabbit	1377	PGSMSKPYHRQGAVNKYVIGWKKSEGSPPPEEPEVPECPGMYSEMDVMSAATQHQTVVGD	1436
				**:::********.********************* *** :*.***:**:**** :****	

Figure S5

A

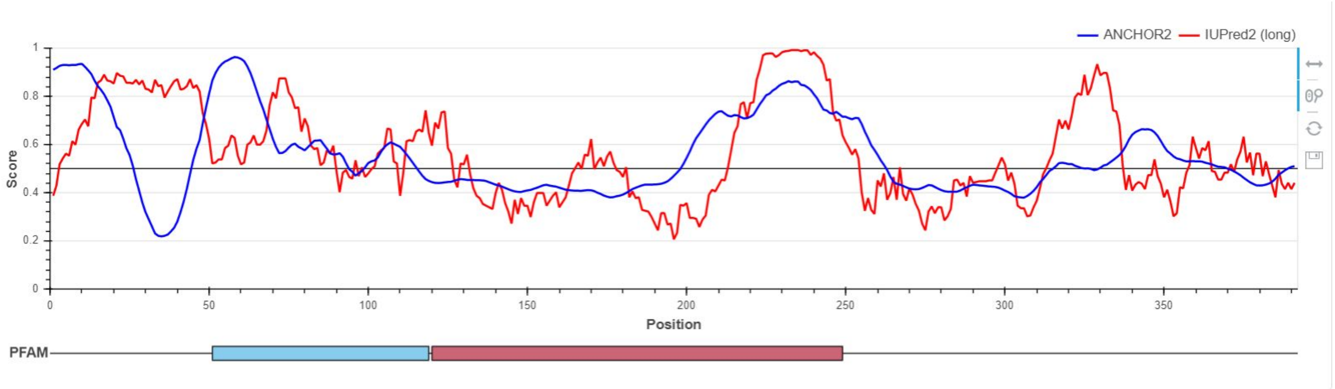

ANCHOR2
IUPred2 (long)
Score
Position
PFAM

B

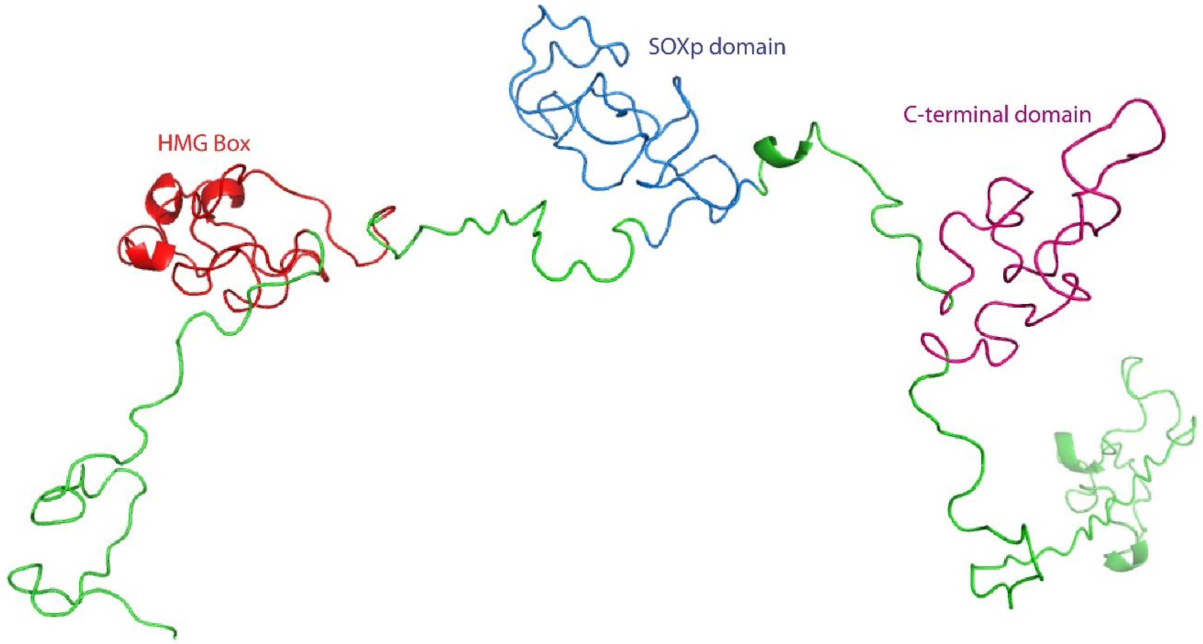

SOXp domain
HMG Box
C-terminal domain

Table S1

Elm Name	Instances (Matched Sequence)	Positions	Elm Description	Cell Compartment	Pattern	Probability
MOD_CK1_1	SEPSGSP	3-9 [A]	CK1 phosphorylation site	cytosol, nucleus	S..([ST])...	1.704e-02
MOD_GlcNHglycan	EPSGS<br>PSGS	4-8 [A]<br>5-8 [A]	Glycosaminoglycan attachment site	extracellular, Golgi apparatus	[ED]{0,3}.(S)[GA].	1.792e-02
MOD_SUMO_for_1	VKSE	1-4 [A]	Motif recognised for modification by SUMO-1	nucleus, PML body	[VILMAFP](K).E	1.914e-03

Table S2

Protein Identified	Start	End	Motif
1. sp|P42684|ABL2_HUMAN	835	843	kKSEeSaaP
2. sp|O96019|ACL6A_HUMAN	111	119	vKSEaSlhP
3. sp|Q8N7Z5|ANR31_HUMAN	44	52	lKSEfSlhP
4. sp|Q9NR48|ASH1L_HUMAN	2842	2850	wKSErSkpP
5. sp|Q9P2D1|CHD7_HUMAN	2249	2257	dKSEeSsqP
6. sp|Q8WTW3|COG1_HUMAN	634	642	gKSEsSekP
7. sp|Q96PL5|ERMAP_HUMAN	416	424	hKSEeSivP
8. sp|Q96PV7|F193B_HUMAN	725	733	kKSEaSpaP
9. sp|Q9UJU5|FOXD3_HUMAN	368	376	iKSEpSarP
10. sp|P20810|ICAL_HUMAN	636	644	kKSEdSkkP
11. sp|O95461|LARG1_HUMAN	204	212	lKSEvSwiP
12. sp|Q5JXC2|MIIP_HUMAN	325	333	pKSEkSsaP
13. sp|P04198|MYCN_HUMAN	350	358	iKSEaSprP
14. sp|P20929|NEBU_HUMAN	6551	6559	eKSEhSeaP
15. sp|P46087|NOP2_HUMAN	614	622	pKSEnSsqP
16. sp|Q14207|NPAT_HUMAN	654	662	sKSEnSqeP
17. sp|Q86W56|PARG_HUMAN	649	657	mKSEySsyP
18. sp|Q684P5|RPGP2_HUMAN	603	611	iKSEtSsnP
19. sp|Q5TZJ5|S31A1_HUMAN	1108	1116	hKSEkSrkP
20. sp|Q5VU36|S31A5_HUMAN	1108	1116	hKSEkSrkP
21. sp|Q5VVP1|S31A6_HUMAN	1104	1112	hKSEkSrkP
22. sp|Q8IWB4|S31A7_HUMAN	1108	1116	hKSEkSrkP
23. sp|Q9P2F8|SI1L2_HUMAN	1401	1409	kKSEgSppP
24. sp|O00570|SOX1_HUMAN	318	326	vKSEpSgsP
25. sp|P48431|SOX2_HUMAN	244	252	vKSEaSssP
26. sp|P41225|SOX3_HUMAN	374	382	vKSEpSspP
27. sp|Q5TCZ1|SPD2A_HUMAN	795	803	sKSEdSelP
28. sp|P49750|YLPM1_HUMAN	910	918	pKSEvSegP
29. sp|P15822|ZEP1_HUMAN	123	131	sKSEeSvsP
30. sp|Q9UL58|ZN215_HUMAN	445	453	sKSEdSnnP
31. sp|Q9NYT6|ZN226_HUMAN	218	226	hKSEkSyrP
32. sp|Q5VUA4|ZN318_HUMAN	1429	1437	eKSEpShlP

Table S3

Entry	Entry name	Protein names	Gene names	Length
Q96PL5	ERMAP_HUMAN	Erythroid membrane-associated protein (hERMAP) (Radin blood group antigen) (Scianna blood group antigen)	ERMAP RD SC	475
P48431	SOX2_HUMAN	Transcription factor SOX-2	SOX2	317
Q8WTW3	COG1_HUMAN	Conserved oligomeric Golgi complex subunit 1 (COG complex subunit 1) (Component of oligomeric Golgi complex 1)	COG1 KIAA1381 LDLB	980
Q5VU36	S31A5_HUMAN	Spermatogenesis-associated protein 31A5 (Protein FAM75A5)	SPATA31A5 FAM75A5	1347
P04198	MYCN_HUMAN	N-myc proto-oncogene protein (Class E basic helix-loop-helix protein 37) (bHLHe37)	MYCN BHLHE37 NMYC	464
Q5TZJ5	S31A1_HUMAN	Spermatogenesis-associated protein 31A1 (Protein FAM75A1)	SPATA31A1 C9orf36 FAM75A1 FAM75A2 SPATA31A2	1347
Q9NYT6	ZN226_HUMAN	Zinc finger protein 226	ZNF226	803
Q5VVP1	S31A6_HUMAN	Spermatogenesis-associated protein 31A6 (Protein FAM75A6)	SPATA31A6 FAM75A6	1343
Q9UL58	ZN215_HUMAN	Zinc finger protein 215 (BWSCR2-associated zinc finger protein 2) (BAZ-2) (Zinc finger protein with KRAB and SCAN domains 11)	ZNF215 BAZ2 ZKSCAN11	517
Q8IWB4	S31A7_HUMAN	Spermatogenesis-associated protein 31A7 (Protein FAM75A7)	SPATA31A7 FAM75A4 FAM75A7 SPATA31A4	1347
Q14207	NPAT_HUMAN	Protein NPAT (Nuclear protein of the ataxia telangiectasia mutated locus) (Nuclear protein of the ATM locus) (p220)	NPAT CAND3 E14	1427
P41225	SOX3_HUMAN	Transcription factor SOX-3	SOX3	446
Q9P2F8	SI1L2_HUMAN	Signal-induced proliferation-associated 1-like protein 2 (SIPA1-like protein 2)	SIPA1L2 KIAA1389	1722
Q5VUA4	ZN318_HUMAN	Zinc finger protein 318 (Endocrine regulatory protein)	ZNF318 HRIHFB2436	2279
P15822	ZEP1_HUMAN	Zinc finger protein 40 (Cirhin interaction protein) (CIRIP) (Gate keeper of apoptosis-activating protein) (GAAP) (Human immunodeficiency virus type I enhancer-binding protein 1) (HIV-EP1) (Major histocompatibility complex-binding protein 1) (MBP-1) (Positive regulatory domain II-binding factor 1) (PRDII-BF1)	HIVEP1 ZNF40	2718
Q9P2D1	CHD7_HUMAN	Chromodomain-helicase-DNA-binding protein 7 (CHD-7) (EC 3.6.4.12) (ATP-dependent helicase CHD7)	CHD7 KIAA1416	2997
P20810	ICAL_HUMAN	Calpastatin (Calpain inhibitor) (Sperm BS-17 component)	CAST	708
O00570	SOX1_HUMAN	Transcription factor SOX-1	SOX1	391
Q5TCZ1	SPD2A_HUMAN	SH3 and PX domain-containing protein 2A (Adapter protein TKS5) (Five SH3 domain-containing protein) (SH3 multiple domains protein 1) (Tyrosine kinase substrate with five SH3 domains)	SH3PXD2A FISH KIAA0418 SH3MD1 TKS5	1133
P49750	YLPM1_HUMAN	YLP motif-containing protein 1 (Nuclear protein ZAP3) (ZAP113)	YLPM1 C14orf170 ZAP3	2146
Q9UJU5	FOXD3_HUMAN	Forkhead box protein D3 (HNF3/FH transcription factor genesis)	FOXD3 HFH2	478
O96019	ACL6A_HUMAN	Actin-like protein 6A (53 kDa BRG1-associated factor A) (Actin-related protein Baf53a) (ArpNbeta) (BRG1-associated factor 53A) (BAF53A) (INO80 complex subunit K)	ACTL6A BAF53 BAF53A INO80K	429
Q96PV7	F193B_HUMAN	Protein FAM193B	FAM193B IRIZIO KIAA1931	902
Q9NR48	ASH1L_HUMAN	Histone-lysine N-methyltransferase ASH1L (EC 2.1.1.43) (ASH1-like protein) (huASH1) (Absent small and homeotic disks protein 1 homolog) (Lysine N-methyltransferase 2H)	ASH1L KIAA1420 KMT2H	2969
P42684	ABL2_HUMAN	Tyrosine-protein kinase ABL2 (EC 2.7.10.2) (Abelson murine leukemia viral oncogene homolog 2) (Abelson tyrosine-protein kinase 2) (Abelson-related gene protein) (Tyrosine-protein kinase ARG)	ABL2 ABLL ARG	1182
Q5JXC2	MIIP_HUMAN	Migration and invasion-inhibitory protein (IGFBP2-binding protein) (Invasion-inhibitory protein 45) (IIp45)	MIIP IIP45	388
Q8N7Z5	ANR31_HUMAN	Ankyrin repeat domain-containing protein 31	ANKRD31	1873
Q86W56	PARG_HUMAN	Poly(ADP-ribose) glycohydrolase (EC 3.2.1.143)	PARG	976
Q684P5	RPGP2_HUMAN	Rap1 GTPase-activating protein 2 (Rap1GAP2) (GTPase-activating Rap/Ran-GAP domain-like protein 4)	RAP1GAP2 GARNL4 KIAA1039 RAP1GA2	730
P20929	NEBU_HUMAN	Nebulin	NEB	6669
P46087	NOP2_HUMAN	Probable 28S rRNA (cytosine(4447)-C(5))-methyltransferase (EC 2.1.1.-) (Nucleolar protein 1) (Nucleolar protein 2 homolog) (Proliferating-cell nucleolar antigen p120) (Proliferation-associated nucleolar protein p120)	NOP2 NOL1 NSUN1	812
O95461	LARG1_HUMAN	LARGE xylosyl- and glucuronyltransferase 1 (EC 2.4.-.-) (Acetylglucosaminyltransferase-like 1A) (Glycosyltransferase-like protein) [Includes: Xylosyltransferase LARGE (EC 2.4.2.-); Beta-1,3-glucuronyltransferase LARGE (EC 2.4.1.-)]	LARGE1 KIAA0609 LARGE	756

Table S4

SNP	coding sequence variant	Missense Variant	Amino Acid Position	Polyphen	SIFT
rs1419694769	A>T	K (Lys) > E (Glu)	K132	probably damaging, score 0.999	deleterious, score 0
rs1389486372	C>T	P (Pro) > S (Ser)	P322	benign, score 0.145	deleterious, score 0.01
	C>A	P (Pro) > T (Thr)		benign, score 0.021	tolerated, score 0.25
rs1178459411	C>A	P (Pro) > T (Thr)	P326	possibly damaging, score 0.813	deleterious, score 0.02
rs1294049725	C>A	P (Pro) > Q (Gln)		probably damaging, score 0.953	tolerated, score 0.08
rs1161821962	C>T	A (Ala) > V (Val)	A328	benign, score 0	tolerated, score 0.46
rs1382937308	C>T	P (Pro) > L (Leu)	P329	benign, score 0	tolerated, score 0.29
rs1362549824	C>T	P (Pro) > S (Ser)		benign, score 0	tolerated, score 1

Table S5

Validation server	HMG domain	SOXp domain	C-terminal domain
**ProSA Web**	-4.01	-2.84	-1.26
**Verify 3D (Residues with an averaged 3D-1D score >= 0.2)**	94.32%	90.91%	71.15%
**Rampage (Residues in favoured/allowed regions)**	100%	100%	100%
